# *MTARC1* and *HSD17B13* Variants Have Protective Effects on Non-Alcoholic Fatty Liver Disease in Patients Undergoing Bariatric Surgery

**DOI:** 10.3390/ijms232415825

**Published:** 2022-12-13

**Authors:** Piotr Kalinowski, Wiktor Smyk, Małgorzata Nowosad, Rafał Paluszkiewicz, Łukasz Michałowski, Bogna Ziarkiewicz-Wróblewska, Susanne N. Weber, Piotr Milkiewicz, Frank Lammert, Krzysztof Zieniewicz, Marcin Krawczyk

**Affiliations:** 1 Department of General, Transplant and Liver Surgery, Medical University of Warsaw, 02-091 Warsaw, Poland; 2 Department of Gastroenterology and Hepatology, Medical University of Gdansk, 80-210 Gdansk, Poland; 3 Department of Pathology, Medical University of Warsaw, 02-091 Warsaw, Poland; 4 Department of Pathology, Warsaw Medical University Clinical Center, 02-091 Warsaw, Poland; 5 Department of Medicine II, Saarland University Medical Center, Saarland University, 66421 Homburg, Germany; 6 Liver and Internal Medicine Unit, Department of General, Transplant and Liver Surgery, Medical University of Warsaw, 02-091 Warsaw, Poland; 7 Hannover Health Science Campus, Hannover Medical School (MHH), 30625 Hannover, Germany; 8 Laboratory of Metabolic Liver Diseases, Department of General, Transplant and Liver Surgery, Centre for Preclinical Research, Medical University of Warsaw, 02-091 Warsaw, Poland

**Keywords:** liver fibrosis, mitochondrial amidoxime-reducing component 1, NAFLD, NASH, weight-loss surgery

## Abstract

The severity of hepatic steatosis is modulated by genetic variants, such as patatin-like phospholipase domain containing 3 (*PNPLA3*) rs738409, transmembrane 6 superfamily member 2 (*TM6SF2*) rs58542926, and membrane-bound O-acyltransferase domain containing 7 (*MBOAT7*) rs641738. Recently, mitochondrial amidoxime reducing component 1 (*MTARC1*) rs2642438 and hydroxysteroid 17-beta dehydrogenase 13 (*HSD17B13*) rs72613567 polymorphisms were shown to have protective effects on liver diseases. Here, we evaluate these variants in patients undergoing bariatric surgery. A total of 165 patients who underwent laparoscopic sleeve gastrectomy and intraoperative liver biopsies and 314 controls were prospectively recruited. Genotyping was performed using TaqMan assays. Overall, 70.3% of operated patients presented with hepatic steatosis. NASH (non-alcoholic steatohepatitis) was detected in 28.5% of patients; none had cirrhosis. The increment of liver fibrosis stage was associated with decreasing frequency of the *MTARC1* minor allele (*p* = 0.03). In multivariate analysis *MTARC1* was an independent protective factor against fibrosis ≥ 1b (OR = 0.52, *p* = 0.03) and ≥ 1c (OR = 0.51, *p* = 0.04). The *PNPLA3* risk allele was associated with increased hepatic steatosis, fibrosis, and NASH (OR = 2.22, *p* = 0.04). The *HSD17B13* polymorphism was protective against liver injury as reflected by lower AST (*p* = 0.04) and ALT (*p* = 0.03) activities. The *TM6SF2* polymorphism was associated with increased ALT (*p* = 0.04). In conclusion, hepatic steatosis is common among patients scheduled for bariatric surgery, but the *MTARC1* and *HSD17B13* polymorphisms lower liver injury in these individuals.

## 1. Introduction

Non-alcoholic fatty liver disease (NAFLD) is currently one of the most common liver diseases worldwide [1]. It is particularly common among obese individuals. Metabolic syndrome or diabetes mellitus represent the major risk factors for the development of a fatty liver [2]. As a result, increased hepatic steatosis is frequently reported in patients undergoing bariatric surgery [3]. Although fatty liver is benign in most affected individuals, it can progress toward liver fibrosis and cirrhosis [4]. The development and progression of NAFLD are mostly attributed to exogenous risk factors, but it is also modulated by genetic predisposition. Previous studies demonstrated that the patatin-like phospholipase domain containing 3 (*PNPLA3*; adiponutrin) p.I148M polymorphism is associated with liver steatosis [5]. Carriers of the minor *PNPLA3* allele are also at risk of liver fibrosis, cirrhosis, and hepatocellular carcinoma (HCC) [6]. In addition, two variants, the transmembrane 6 superfamily member 2 *(TM6SF2*) p.E167K and membrane bound O-acyltransferase domain containing 7 (*MBOAT7*) p.G17E were previously linked to progressive NAFLD [7,8]. *MBOAT7* is involved in the regulation of intracellular arachidonic acid [9], whereas carriers of the *TM6SF2* polymorphisms have increased liver fat content but lower serum levels of triglycerides and cholesterol [10].

Recently, two common polymorphisms were shown to have protective effects in NAFLD. Indeed, carriers of the hydroxysteroid 17-beta dehydrogenase 13 (*HSD17B13)* rs72613567 and mitochondrial amidoxime reducing component 1 (*MTARC1*) p.A165T variants might have lower hepatic injury in the setting of chronic liver diseases. Abul-Husn et al. [11] analyzed exome sequence data and electronic health records from participants in the DiscovEHR human genetics study as well as 2391 human liver biopsies and demonstrated that in homozygous and heterozygous carriers of the *HSD17B13* variant, the risks of NAFLD and NASH-cirrhosis were reduced by 30% and 17%, and 49% and 26%, respectively. The *HSD17B13* variant ameliorated liver injury associated with the *PNPLA3* risk allele [11]. The second protective variant, *MTARC1*, was shown to have beneficial effects on hepatic steatosis and cirrhosis and to correlate with improved plasma liver tests and lipoprotein profile [12]. Our candidate gene study demonstrated the protective effects of *MTARC1* p.A165T polymorphism on liver injury in patients with autoimmune hepatitis (AIH) [13]. A brief report from Luukkonen et al. highlighted that individuals carrying the minor allele of the *MTARC1* p.A165T polymorphism are characterized by higher blood phosphatidylcholine levels and decreased parameters of NAFLD severity as compared to carriers of the wild-type *MTARC1* genotype [14]. Furthermore, the *MTARC1* minor allele was also associated with a reduced risk of alcohol-related cirrhosis [15] and liver-related mortality [16].

Our current study analyzes a cohort of 165 prospectively recruited Caucasian Polish patients who underwent liver biopsy during weight loss surgery. In all patients, we genotyped the five mentioned variants and analyzed them in relation to liver biopsies and to the patient’s clinical data.

## 2. Results

Detailed baseline characteristics of the study cohort are presented in Table 1. We recruited 165 patients (median age 42 years, 66.7% women). Their mean body mass index (BMI) was 43.8 ± 5.7 kg/m^2^, and 48 (29.1%) had type 2 diabetes mellitus. The control cohort comprised 314 adult individuals without chronic liver diseases (median age 62 years, 68.3% women). A liver biopsy was performed intraoperatively in all patients undergoing bariatric surgery. In total, 116 patients in our cohort had NAFLD (i.e., steatosis ≥5%). Patients with NAFLD were characterized by significantly (Table 1) higher serum aspartate aminotransferase (AST), alanine aminotransferase (ALT), and gamma-glutamyl transferase (GGT) activities as compared to patients without NAFLD. Moreover, the presence of NAFLD was associated with higher triglycerides, homeostatic model assessment for insulin resistance (HOMA-IR), and lower high-density lipoprotein (HDL) cholesterol levels.

As shown in Table 2, 70.3% of included patients had hepatic steatosis, NASH was present in 28.5% of operated patients, 2.4% had liver fibrosis F3, and none of them had liver cirrhosis. All five selected variants were successfully genotyped in 165 patients and 314 controls included in the study. The distribution of all genotyped variants is presented in Table 3. Each variant was within the HWE, which underscores robust genotyping. Comparison of the *PNPLA3* risk allele frequencies between patients undergoing bariatric surgery and controls demonstrated a significantly (*p* < 0.01) lower frequency of the minor allele in the latter group.

Comparison of the *PNPLA3* risk allele frequencies between patients undergoing bariatric surgery and controls demonstrated a significantly (*p* < 0.01) lower frequency of the minor allele in the latter group.

**Figure 1:**
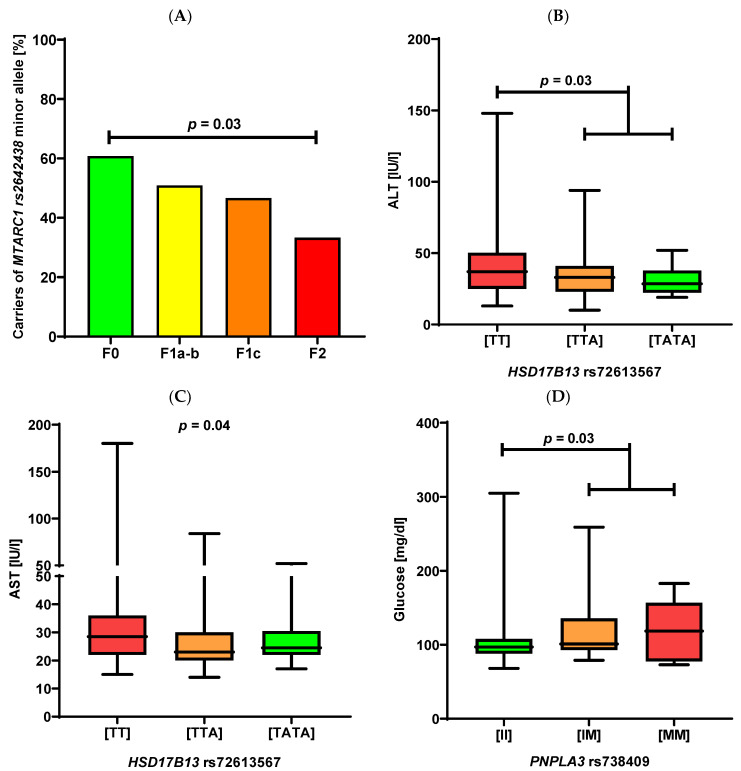

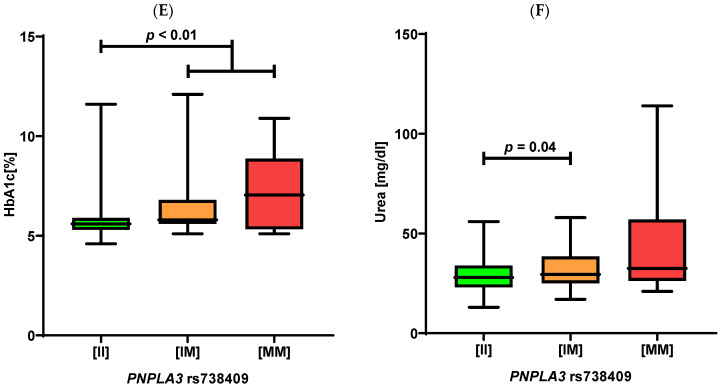
(**A**): Distribution of the *MTARC1* minor allele in relation to liver fibrosis at liver biopsy in individuals undergoing bariatric surgery. F0 n = 31, F1a–b n = 29, F1c n = 14, F2 n = 7. (**B**,**C**): Association between the *HSD17B13* polymorphism and serum ALT (**B**) and AST activities (**C**). Genotype frequencies: [TT] n = 102, [TTA] n = 51, [TATA] n = 12. (**D**–**F**): Association between the *PNPLA3* variant and glucose (**D**), HbA1c (**E**), and urea (**F**) levels. Genotype frequencies: [II] n = 117, [IM] n = 42, [MM] n = 6.

As shown in Figure 1A, the increment of liver fibrosis was associated with decreasing frequency of the *MTARC1* minor allele (*p* = 0.03). The second protective genetic variant, namely *HSD17B13*, influenced serum ALT and AST activities. As shown in Figure 1B,C, carriers of the *HSD17B13* polymorphism had significantly lower serum ALT (*p* = 0.03, Figure 1B) and AST activities (*p* = 0.04, Figure 1C). Carriers of the *TM6SF2* variant presented with increased serum ALT activity (*p* = 0.04).

We included the non-genetic risk factors and tested variants as covariates in regression models to analyze the determinants of liver steatosis and fibrosis. The results are presented in Table 4 and Table 5 (univariate analysis left panel, multivariate analysis right panel). In the multivariate model, variant *MTARC1* was an independent protective factor against liver fibrosis ≥ 1b (OR = 0.52, 95% CI 0.29–0.92; *p* = 0.03) and ≥1c (OR = 0.51, 95% CI 0.28–0.92; *p* = 0.04). On the other hand, *PNPLA3* and BMI represented independent risk factors for fibrosis stage ≥ 2 (OR = 3.09, 95% CI 1.49–6.40; *p* = 0.002; OR = 1.12, 95% CI 1.04–1.21; *p* = 0.003, respectively). In the multivariate models, the *PNPLA3* represented a risk factor for steatosis grade ≥2 (OR = 2.27, 95% CI 1.24–4.15; *p* = 0.008) and grade 3 (OR = 3.69, 95% CI 1.56–8.70; *p* = 0.003). The *TM6SF2* variant was associated with the risk for advanced steatosis (grade S3) (OR = 6.19, *p* = 0.001, Table 4). In the subgroup of *PNPLA3* risk allele carriers (N = 48), regression analysis showed an association of the *MTARC1* polymorphism with significantly reduced risk of fibrosis stage ≥ 1a (OR = 0.16; *p* = 0.02) and stage ≥ 1b (OR = 0.29; *p* = 0.03). None of the analyzed variants correlated significantly with liver inflammation or hepatocyte ballooning. 

We performed regression analyses to detect risk factors for NASH in our cohort. The results are presented in Table 6. In the univariate model, we detected a trend for protection against NASH conferred by the *MTARC1* polymorphism (*p* = 0.08), whereas the *PNPLA3* variant, hyperlipidemia, and diabetes were all associated with a significantly increased risk of NASH. In a multivariate model including the *PNPLA3* variant, hyperlipidemia, and diabetes, only the first two proved to represent independent risk factors for non-alcoholic steatohepatitis (Table 6).

Concerning the metabolic profiles in our patients, we detected significant associations between the *PNPLA3* p.I148M and increased fasting glucose (*p* = 0.03; Figure 1D), as well as, HbA1c levels (*p* < 0.01, Figure 1E). This polymorphism was also associated with an increased risk of developing type 2 diabetes (OR = 2.35, 95% CI 1.09–3.87; *p* = 0.03) and higher blood urea concentrations (*p* = 0.04, Figure 1F). Finally, we did not detect any association between patients’ phenotypes and the *MBOAT7* polymorphism.

## 3. Discussion

Our current manuscript presents a comprehensive analysis of the genetic variants associated with NAFLD in patients undergoing bariatric surgery. To the best of our knowledge, this is one of the first such studies on individuals coming from Eastern Europe. In line with the previous reports, we detected harmful effects of the *PNPLA3* p.I148M polymorphism on liver phenotypes and metabolic profiles in recruited patients. We also demonstrate that the common missense variant of *MTARC1* p.A165T is protective against hepatic fibrosis in obese individuals and ameliorates the harmful effects of the *PNPLA3* p.I148M minor allele. The protection conferred by the *HSD17B13* rs72613567 polymorphism was less pronounced. It was associated with lower ALT and AST activities but not with the liver biopsy results.

We detected protective effects of the *MTARC1* p.A165T polymorphism in our cohort. Luukkonen et al. reported previously that the *MTARC1* variant is associated with a low SAF (steatosis-activity-fibrosis) score and decreased lobular inflammation, activity, and fibrosis [14]. Similar to our findings, the *MTARC1* genotype did not correlate with steatosis in that study [14]. In our cohort, the protective effect of *MTARC1* p.A165T minor allele on liver fibrosis was documented despite the absence of patients with cirrhosis, which could further increase the significance of the effect. Emdin et al. performed a large multicohort study and identified *MTARC1* p.A165T as a protective variant against NAFLD-related cirrhosis and all-cause cirrhosis [12]. This variant was also associated with reduced hepatic steatosis diagnosed by physicians or assessed by imaging studies such as computed tomography [12] and magnetic resonance [17]. The decreased risk of developing NAFLD due to the *MTARC1* variant was also confirmed in a recent analysis of 9491 cases with fatty liver [18]. MTARC1 is an enzyme located in the outer mitochondrial membrane that counterparts with other enzymatic systems and catalyzes the reduction of N-hydroxylated prodrugs, N-oxygenated compounds, and N(omega)-hydroxy-L-arginine [19,20]. The protective *MTARC1* variant has been predicted by PolyPhen-2 to be deleterious to MTARC1 protein function [15]. Therefore, the reduced risk of liver injury associated with this polymorphism seems to originate from the loss of *MTARC1* function.

HSD17B13 has a function of retinol dehydrogenase targeted to hepatic lipid droplets [21]. In our cohort, we did not detect any significant association between *HSD17B13* variant and liver histology. However, lower activities of transaminases in carriers of the *HSD17B13* polymorphism point to its potential protective effects. This observation is in line with the latest genome-wide association study (GWAS) by Gao et al. in Europeans, demonstrating that *HSD17B13* and the *MTARC1* and *PNPLA3* polymorphisms substantially modulate serum transaminases [22]. The findings reported in our manuscript are partially in line with the GWAS by Anstee et al. [23]. This study demonstrated an association between the increased risk of biopsy-confirmed NAFLD and *PNPLA3* as well as *TM6SF2* variants and a trend towards a lower NAFLD risk in carriers of the *HSD17B13* polymorphism.

Eventually, we confirmed that the *PNPLA3* p.I148M polymorphism negatively affects the patients’ liver status. Indeed, not only was it associated with the NAFLD stage but also with a worse metabolic profile in analyzed patients. Interestingly, we detected a lower frequency of the *PNPLA3* variant in patients scheduled for bariatric surgery compared to controls. We speculate that obese carriers of this variant might be less frequently scheduled for bariatric surgery due to their health status, but this needs to be evaluated in additional studies. Variant *PNPLA3* p.I148M is by far the most frequently investigated and replicated genetic modifier of liver injury in NAFLD [5,24]. An example of the robust association between the *PNPLA3* p.I148M variant and NAFLD severity was demonstrated in GWAS in the population of European ancestry [25]. Carriers of the minor *PNPLA3* allele are known to be at risk of the entire spectrum of progressive liver steatosis from NAFLD to NASH, fibrosis, cirrhosis, and hepatocellular carcinoma [26]. Functional studies demonstrated that PNPLA3 is a lipase [27]. Alternatively, its retinyl-palmitate lipase activity in hepatic stellate cells was described [28]. *PNPLA3* p.I148M and *TM6SF2* p.E167K were associated with increased hepatic steatosis in our cohort. In contrast to *PNPLA3*, *TM6SF2* did not affect fibrosis. This is in line with our previous studies in German and Polish patients with fatty liver [29,30]. *TM6SF2* p.E167K was associated in both cohorts with hepatic steatosis, but a biopsy-based analysis of liver fibrosis in the German cohort did not yield a significant association between this variant and liver scarring [29]. In a large cohort study, Stender et al. found that adiposity amplified the genetic risk of NAFLD associated with SNPs (including *PNPLA3* and *TM6SF2*) [31]. The effect of risk variants on hepatic steatosis assessed by magnetic resonance increased with BMI and was the greatest in severely obese individuals (BMI > 35 kg/m^2^). Hence, the risk of NAFLD development and progression at different stages, from steatosis to cirrhosis, is affected by the additive interaction between genetic variants and adiposity [31].

In our previous MRI-based analysis of Spanish patients undergoing bariatric surgery [32], we demonstrated that the *PNPLA3*, but not *TM6SF2* or *MBOAT7* variants, is associated with a greater reduction of hepatic fat content within 12 months after surgery. Overall, these results underscore the notion that obese individuals carrying the *PNPLA3* minor allele are, on the one hand, at risk of a rapid progression of NAFLD, and they might be the ones who, in particular, profit from weight-loss therapy and lifestyle modifications [33]. Our findings suggest the possible reduction of detrimental *PNPLA3* effects in obese individuals by *MTARC1*, but a small subgroup size is a limitation and requires confirmation. One possible solution is to consider *MTARC1* p.A165T in assessing an individual’s risk and include it in a polygenic risk score. Such risk scores based on combinations of genetic risk variants have been published recently and showed significant associations with NAFLD severity [23,29]. In the future, we can expect novel NAFLD-risk modifiers that may emerge as more data is published from studies employing techniques such as GWAS or quantitative trait locus (QTL) mapping [25,34].

In conclusion, we demonstrate that *MTARC1* p.A165T, and to a lesser extent, *HSD17B13* rs72613567 polymorphism can be protective against NAFLD-related liver injury in patients with obesity scheduled for bariatric surgery. In particular, *MTARC1* p.A165T can lower the harmful effects of the *PNPLA3* p.I148M which remains the central risk factor for a progressive NAFLD. Since the function of MTARC1 is unclear, further studies in patients with chronic liver disease aimed at explaining the association between this genotype and clinical presentation are needed.

## 4. Materials and Methods

### 4.1. Study Cohort and Clinical Data

All patients were recruited at the Medical University of Warsaw. The study protocols (KB/140/2015 and KB/237/2015) were approved by the local ethics committee according to the ethical guidelines of the 1975 Declaration of Helsinki (latest revision, 2013), and written and informed consent was obtained from all participants. Inclusion criteria were based on clinical indications for bariatric surgery [35]. The criteria were fulfilled by 172 patients who were further evaluated and underwent laparoscopic sleeve gastrectomy and intraoperative liver biopsy. A group of adult controls without chronic liver diseases was recruited from outpatients in our hospital. A wedge liver biopsy was taken from the left lobe of the liver. Liver specimens collected intraoperatively were evaluated according to the NAFLD Activity Score (NAS) [36], which equals a sum of unweighted scores of steatosis (0–3), lobular inflammation (0–3), and ballooning (0–2). The NAS ≥ 5 corresponds with NASH. The stage of fibrosis was scored 0, 1a, 1b, 1c, 2, 3, and 4, as follows: 0—none; 1a—mild, zone 3, perisinusoidal; 1b—moderate, zone 3, perisinusoidal; 1c—portal/periportal; 2—perisinusoidal and portal/periportal; 3—bridging fibrosis; 4—cirrhosis. Exclusion criteria included previous history or positive serum markers of chronic liver disease. After the exclusion of 7 patients (4 positive for viral hepatitis, 3 with a history of alcohol abuse), 165 patients were finally included in the study.

All patients underwent clinical examination. Blood samples were drawn from fasted subjects, and liver function tests were determined by standard clinical-chemical assays in the central laboratory of our center.

### 4.2. Genotyping

DNA was extracted from peripheral blood mononuclear cells using the DNeasy Blood & Tissue Kit (Qiagen). DNA concentrations were measured using a NanoDrop spectrophotometer. Genotyping of the *MTARC1* p.A165T (rs2642438), *PNPLA3* p.I148M (rs738409), as well as other variants associated with the risk of NAFLD, namely *TM6SF2* p.E167K (rs58542926), *MBOAT7* p.G17E (rs641738), and *HSD17B13* (rs72613567), was performed using TaqMan assays as described previously [25]. The fluorescence data were analyzed with allelic discrimination 7500 Software v.2.0.2.

### 4.3. Statistical Analyses

Statistical analyses were performed using SPSS (version 26.0; SPSS, Munich, Germany) and GraphPad Prism (version 8.0; GraphPad Software, San Diego, CA, USA). The nominal *p*-value < 0.05 was regarded as statistically significant. The consistency of genotyping distributions with the Hardy–Weinberg equilibrium (HWE) was tested by exact tests (https://ihg.helmholtz-muenchen.de/cgi-bin/hw/hwa1.pl accessed on 9 November 2022). The Shapiro–Wilk test was used to determine whether the set of observations followed normal distributions. Student t and Mann–Whitney-U tests were used to study normally and non-normally distributed parameters, respectively. The effects of genetic variants, age, BMI, and sex on patients’ phenotypes were tested by univariate and multivariate regression analyses.

## Figures and Tables

**Table 1:** Baseline characteristics of the study cohort.

	Entire Cohort	Patients without NAFLD	Patients with NAFLD
**Total participants, n**	165	49 (29.7%)	116 (70.3%)
**Female, n [%]**	110 (66.7%)	39 (79.6%)	71 (61.2%)
**Age [years]**	42 (19–65)	39 (19–63)	42 (21–65)
**BMI [kg/m^2^]**	43.8 (34.2–64.3)	43.8 (34.2–56.0)	43.7 (34.3–64.3)
**Hypertension**	107 (64.8%)	25 (51.0%)	82 (70.7%)
**Type 2 diabetes mellitus**	48 (29.1%)	4 (8.2%)	44 (37.9%)
**Hyperlipidaemia**	109 (66.1%)	23 (46.9%)	86 (74.1%)
**Haemoglobin [g/dL]**	14.1 (10.2–18.3)	13.6 (10.2–18.3)	14.2 (10.6–18.0)
**Platelets [G/L]**	258.0 (122.0–426.0)	266.0 (122.0–426.0)	253.0 (131.0–425.0)
**Creatinine [mg/dL]**	0.8 (0.5–3.6)	0.8 (0.6–3.6)	0.8 (0.5–2.1)
**Urea [mg/dL]**	29.0 (13.0–114.0)	28.0 (13.0–114.0)	29.0 (14.0–58.0)
**ALT [IU/L]**	34.0 (10.0–148.0)	25.0 (13.0–94.0)	38.0 (10.0–148.0)
**AST [IU/L]**	26.0 (14.0–180.0)	22.0 (15.0–108.0)	29.0 (14.0–180.0)
**ALP [IU/L]**	72.0 (25.0–133.0)	73.0 (40.0–116.0)	71.0 (25.0–133.0)
**GGT [IU/L]**	31.0 (12.0–296.0)	23.0 (12.0–192.0)	35.5 (12.0–296.0)
**Bilirubin [mg/dL]**	0.6 (0.1–1.9)	0.5 (0.2–1.7)	0.6 (0.1–1.9)
**Amylase [IU/L]**	38.5 (17.0–144.0)	37.0 (18.0–144.0)	39.0 (17.0–89.0)
**Lipase [IU/L]**	26.0 (13.0–180.0)	25.0 (14.0–86.0)	29.0 (13.0–180.0)
**Total cholesterol [mg/dL]**	185.0 (97.0–273.0)	176.0 (97.0–270.0)	187.0 (115.0–273.0)
**Triglycerides [mg/dL]**	151.0 (58.0–779.0)	117.0 (58.0–263.0)	163.5 (63.0–779.0)
**HDL [mg/dL]**	44.0 (18.0–88.0)	47.5 (29.0–88.0)	42.0 (18.0–84.0)
**LDL [mg/dL]**	105.0 (27.4–213.0)	104.0 (29.0–213.0)	105.0 (27.4–181.0)
**FIB-4 [points]**	0.7 (0.2–5.0)	0.7 (0.2–2.1)	0.8 (0.3–5.0)
**NFS [points]**	−0.85 (−4.0–4.8)	−1.1 (−3.0–2.7)	−0.7 (−4.0–4.8)
**Glycaemia [mg/dL]**	97.0 (68.0–305.0)	92.0 (73.0–126.0)	101.0 (68.0–305.0)
**HbA1c [%]**	5.7 (4.6–12.1)	5.4 (4.9–7.1)	5.8 (4.6–12.1)
**C-peptide [ng/mL]**	3.8 (0.7–11.8)	3.1 (2.0–5.5)	3.9 (0.7–11.8)
**Insulin [IU/mL]**	19.7 (4.8–177.0)	14.5 (6.1–79.4)	21.7 (4.8–177.0)
**HOMA-IR [points]**	4.7 (1.0–74.0)	3.4 (1.2–14.3)	5.5 (1.0–74.0)

Values are presented as medians (ranges). Abbreviations: ALP, alkaline phosphatase; ALT, alanine aminotransferase; AST, aspartate aminotransferase; BMI, body mass index; FIB-4, fibrosis-4 score; GGT, Gamma-glutamyl transferase; HbA1c, glycated hemoglobin; HDL, High-density lipoprotein; HOMA-IR, homeostatic model assessment for insulin resistance; LDL, low-density lipoprotein; NAFLD, non-alcoholic fatty liver disease; NFS, NAFLD fibrosis score.

**Table 2:** Results of liver biopsies in 165 patients undergoing bariatric surgery.

Histology	Score	Number of Patients	%
**Steatosis**			
<5%	0	49	29.7
5–33%	1	64	38.8
>33–66%	2	36	21.8
>66%	3	16	9.7
**Lobular inflammation**			
No foci	0	15	9.1
<2 foci/200×	1	69	41.8
2–4 foci/200×	2	71	43.0
>4 foci/200×	3	10	6.1
**Hepatocyte ballooning**			
None	0	48	29.1
Few balloon cells	1	77	46.7
Many cells/prominent ballooning	2	40	24.2
**NAS**			
0–2	0–2	51	30.9
3–4	3–4	67	40.6
≥5	≥5	47	28.5
**Fibrosis**			
None	0	51	30.9
Mild, zone 3, perisinusoidal	1a	57	34.6
Moderate, zone 3, perisinusoidal	1b	2	1.2
Portal/periportal	1c	30	18.2
Perisinusoidal and portal/periportal	2	21	12.7
Bridging fibrosis	3	4	2.4
Cirrhosis	4	0	0

**Table 3:** Genotype frequencies in all patients scheduled for bariatric surgery (cases) and in controls.

Cases					
	*MTARC1* (rs2642438)	*PNPLA3* (rs738409)	*TM6SF2* (rs58542926)	*MBOAT7* (rs641738)	*HSD17B13* (rs72613567)
**Wild-type**	80 (48.5%)	117 (70.9%)	149 (90.3%)	56 (33.9%)	102 (61.8%)
**Heterozygous variant**	75 (45.4%)	42 (25.5%)	14 (8.5%)	81 (49.1%)	51 (30.9%)
**Homozygous variant**	10 (6.1%)	6 (3.6%)	2 (1.2%)	28 (17.0%)	12 (7.3%)
**Controls**					
**Wild-type**	144 (45.9%)	181 (57.6%)	278 (88.5%)	90 (28.6%)	176 (56.1%)
**Heterozygous variant**	142 (45.2%)	114 (36.3%)	35 (11.2%)	166 (52.9%)	110 (35.0%)
**Homozygous variant**	28 (8.9%)	19 (6.1%)	1 (0.3%)	58 (18.5%)	28 (8.9%)
***p***	0.37	<0.01	0.79	0.30	0.23
**OR**	0.85	0.64	1.06	0.87	0.84

The differences in genotype distributions between cases and controls were analyzed using Armitage’s trend test. Abbreviations: *HSD17B13*, 17β-Hydroxysteroid dehydrogenase type 13; *MTARC1*, mitochondrial amidoxime-reducing component 1; *MBOAT7*, membrane-bound O-acyltransferase domain-containing protein 7; OR, odds ratio; *PNPLA3*, patatin-like phospholipase domain-containing protein 3 also known as adiponutrin; *TM6SF2*, transmembrane 6 superfamily 2 human genes.

**Table 4:** Risk and protective factors for developing liver fibrosis.

	Univariate Analysis		Multivariate Analysis	
Factor	Odds Ratio (95% CI)	*p*	Odds Ratio (95% CI)	*p*
**Fibrosis Grade ≥ 1a**				
***MTARC1* (rs2642438)**	**0.56 (0.32–0.96)**	**0.04**	NA	
*PNPLA3* (rs738409)	1.65 (0.84–3.23)	0.15	NA	
*TM6SF2* (rs58542926)	1.50 (0.52–4.35)	0.45	NA	
*MBOAT7* (rs641738)	0.96 (0.60–1.55)	0.87	NA	
*HSD17B13* (rs72613567)	1.37 (0.79–2.39)	0.26	NA	
BMI (kg/m^2^)	1.01 (0.95–1.07)	0.74	NA	
Age (years)	1.00 (0.97–1.04)	0.95	NA	
Gender	0.77 (0.37–1.58)	0.48	NA	
**Fibrosis Grade ≥ 1b**				
***MTARC1* (rs2642438)**	**0.55 (0.31–0.96)**	**0.04**	**0.52 (0.29–0.92)**	**0.03**
*PNPLA3* (rs738409)	1.75 (0.98–3.13)	0.06	NA	
*TM6SF2* (rs58542926)	1.79 (0.73–4.35)	0.20	NA	
*MBOAT7* (rs641738)	1.16 (0.73–1.84)	0.53	NA	
*HSD17B13* (rs72613567)	1.08 (0.65–1.79)	0.78	NA	
BMI (kg/m^2^)	1.03 (0.98–1.09)	0.25	NA	
**Age (years)**	**1.04 (1.00–1.08)**	**0.03**	**1.04 (1.01–1.08)**	**0.02**
Gender	0.70 (0.36–1.37)	0.30	NA	
**Fibrosis Grade ≥ 1c**				
***MTARC1* (rs2642438)**	**0.55 (0.31–0.97)**	**0.04**	**0.51 (0.28–0.92)**	**0.04**
***PNPLA3* (rs738409)**	**1.87 (1.04–3.35)**	**0.04**	1.79 (0.98–3.30)	0.06
*TM6SF2* (rs58542926)	1.88 (0.77–4.58)	0.17	NA	
*MBOAT7* (rs641738)	1.14 (0.72–1.82)	0.58	NA	
*HSD17B13* (rs72613567)	1.07 (0.64–1.79)	0.79	NA	
BMI (kg/m^2^)	1.04 (0.98–1.10)	0.18	NA	
**Age (years)**	**1.04 (1.00–1.07)**	**0.04**	1.04 (1.00–1.08)	0.06
Gender	0.72 (0.37–1.43)	0.35	NA	
**Fibrosis Grade ≥ 2**				
*MTARC1* (rs2642438)	0.54 (0.25–1.17)	0.12	NA	
***PNPLA3* (rs738409)**	**2.86 (1.43–5.72)**	**0.003**	**3.09 (1.49–6.40)**	**0.002**
*TM6SF2* (rs58542926)	2.56 (0.97–6.72)	0.06	NA	
*MBOAT7* (rs641738)	1.13 (0.61–2.08)	0.70	NA	
*HSD17B13* (rs72613567)	0.64 (0.29–1.38)	0.25	NA	
**BMI (kg/m^2^)**	**1.11 (1.04–1.20)**	**0.003**	**1.12 (1.04–1.21)**	**0.003**
Age (years)	1.02 (0.97–1.06)	0.46	NA	
Gender	0.58 (0.25–1.39)	0.22	NA	

NA—not applied; only statistically significant factors from the univariate analysis were included in the multivariate analysis. Abbreviations: see Table 1 and Table 3.

**Table 5:** Risk and protective factors for developing liver steatosis.

	Univariate Analysis		Multivariate Analysis	
Factor	Odds Ratio (95% CI)	*p*	Odds Ratio (95% CI)	*p*
**Steatosis grade ≥ S1**				
*MTARC1* (rs2642438)	1.20 (0.68–2.09)	0.53	NA	
*PNPLA3* (rs738409)	1.38 (0.71–2.65)	0.34	NA	
*TM6SF2* (rs58542926)	1.98 (0.60–6.58)	0.26	NA	
*MBOAT7* (rs641738)	1.25 (0.77–2.04)	0.37	NA	
*HSD17B13* (rs72613567)	0.77 (0.46–1.29)	0.31	NA	
BMI (kg/m^2^)	1.01 (0.95–1.07)	0.81	NA	
Age (years)	1.02 (0.98–1.05)	0.35	NA	
**Gender**	**0.41 (0.18–0.89)**	**0.03**	NA	
**Steatosis grade ≥ S2**				
*MTARC1* (rs2642438)	0.68 (0.38–1.19)	0.17	NA	
***PNPLA3* (rs738409)**	**2.27 (1.25–4.12)**	**0.007**	**2.27 (1.24–4.15)**	**0.008**
***TM6SF2* (rs58542926)**	**2.51 (1.00–6.28)**	**0.049**	2.51 (0.98–6.46)	0.056
*MBOAT7* (rs641738)	1.25 (0.78–2.01)	0.36	NA	
*HSD17B13* (rs72613567)	0.89 (0.52–1.51)	0.66	NA	
BMI (kg/m^2^)	1.01 (0.95–1.07)	0.82	NA	
Age (years)	1.02 (0.98–1.05)	0.33	NA	NA
Gender	0.72 (0.36–1.43)	0.34	NA	
**Steatosis grade = S3**				
*MTARC1* (rs2642438)	1.65 (0.73–3.75)	0.23	NA	
***PNPLA3* (rs738409)**	**3.46 (1.55–7.72)**	**0.002**	**3.69 (1.56–8.70)**	**0.003**
***TM6SF2* (rs58542926)**	**5.66 (1.99–16.11)**	**0.001**	**6.19 (2.00–19.17)**	**0.001**
*MBOAT7* (rs641738)	1.28 (0.61–2.67)	0.52	NA	
*HSD17B13* (rs72613567)	0.95 (0.41–2.19)	0.91	NA	
BMI (kg/m^2^)	1.07 (0.99–1.17)	0.11	NA	
Age (years)	1.00 (0.95–1.05)	0.96	NA	
Gender	0.82 (0.28–2.38)	0.71	NA	

NA—not applied; only statistically significant factors from the univariate analysis were included in the multivariate analysis. Abbreviations: see Table 1 and Table 3.

**Table 6:** Risk and protective factors for NASH.

	Univariate Analysis		Multivariate Analysis	
Factor	Odds Ratio (95% CI)	*p*	Odds Ratio (95% CI)	*p*
*MTARC1* (rs2642438)	0.60 (0.33–1.08)	0.08	NA	
***PNPLA3* (rs738409)**	**2.26 (1.24–4.13)**	**0.008**	**2.22 (1.03–4.80)**	**0.04**
*TM6SF2* (rs58542926)	2.32 (0.94–5.75)	0.07	NA	
*MBOAT7* (rs641738)	1.06 (0.65–1.72)	0.81	NA	
*HSD17B13* (rs72613567)	1.05 (0.61–1.79)	0.86	NA	
**Type 2 diabetes mellitus**	**8.94 (3.94–20.28)**	**<0.001**	**7.91 (3.32–18.86)**	**<0.001**
**Hyperlipidemia**	**2.68 (1.14–6.30)**	**0.02**	2.93 (0.97–8.89)	0.06
BMI (kg/m^2^)	1.01 (0.95–1.07)	0.75	NA	
Age (years)	1.01 (0.98–1.05)	0.57	NA	
Gender	1.09 (0.53–2.25)	0.81	NA	

NA—not applied; only statistically significant factors from the univariate analysis were included in the multivariate analysis. Abbreviations: see Table 1 and Table 3; NASH, non-alcoholic steatohepatitis.

## Data Availability

All data generated or analyzed during this study are included in this article. Further inquiries can be directed to the corresponding author.
